# Magnitude of Severe Acute Maternal Morbidity and Associated Factors Related to Abortion: A Cross-Sectional Study in Hawassa University Comprehensive Specialized Hospital, Ethiopia, 2019

**DOI:** 10.1155/2020/1781652

**Published:** 2020-12-19

**Authors:** Mequanent Tariku

**Affiliations:** Debre Tabor University, College of Health Science, Debre Tabor, Ethiopia

## Abstract

**Background:**

Abortion-related mortality is decreasing, but the complication is still causing a significant morbidity to mothers especially in developing countries. Recently, suitable criteria to assess maternal near miss for sub-Saharan countries were adapted in harmony with the previous World Health Organization near-miss criteria. The aim of this study was to assess the magnitude of severe acute maternal morbidity and associated factors related to abortion in Hawassa University Comprehensive Specialized Hospital, Ethiopia.

**Method:**

An institution-based cross-sectional study was conducted among 337 women who sought abortion services at Hawassa University Comprehensive Specialized Hospital from January 1 to October 30, 2019. The participants were selected conveniently. Data was collected by using prospective morbidity methodology with pretested anonymous structured English questionnaire. The collected data were then entered into SPSS version 20 for analysis. Variables with *p* value ≤ 0.2 in the bivariate analysis, not collinear, were entered to multivariable regression. The strength of association is presented by odds ratio and 95% confidence interval. *p* value less than 0.05 was used as a cut-off point to determine statistically significant association.

**Results:**

The magnitude of severe acute maternal morbidity and maternal near miss is found to be 35.6 and 17.7%, respectively. Factors significantly associated with severe acute maternal morbidity were as follows: women uneducated (AOR: 3.02; 95% CI 1.24-7.33), second-trimester pregnancy (1.89-9.14), and delayed presentation (AOR: 4.32, 95% CI 1.76-10.59).

**Conclusion:**

Severe acute maternal morbidity and maternal near miss related to abortion are high despite the availability of safe termination. Near-miss cases could be better traced by using reasonably adapted World Health Organization near-miss criteria for sub-Saharan countries. Lack of education, increased gestational age, and delayed presentation had increased severe acute maternal morbidity associated with abortion which may need further education on health care seeking culture of the community.

## 1. Introduction

Maternal mortality due to abortion is decreasing both worldwide and in Ethiopia [1–3]. During 1980-1999, abortion-related complication was the leading cause of maternal mortality in Ethiopia, accounting for 31%. This steadily decreased to 10% during the period 2000-2012 [2].

However, severe morbidity from abortion complications remains high [4–7]. Measuring morbidity becomes one of the health outcome indicators which show the progress of health care service [8]. Severe acute maternal morbidity (SAMM) is a good measurement to ascertain the burden of unsafe abortion [4].

A cross-sectional study published in 2017 in Zambia district provincial and tertiary hospitals over 5 months on incidence of abortion-related near-miss complication shows that near miss constituted 16% of admitted complications. This study included the WHO's criteria and the adapted near-miss criteria for sub-Saharan countries [9].

A cross-sectional study of abortion complication severity and associated factors, done in Kenya, showed severe morbidity in 37.1% of women. In this national study, gestational age more than 12 weeks and delayed presentation had a higher proportion of moderate or severe postabortion complication (*p* < 0.05). History of interference and unwanted pregnancy had a significant association with severe complications (*p* < 0.001) [10].

A national estimate in Ethiopia concerning abortion complications in 2010 shows severe morbidity in 27% [6]. In 2008 and 2014, severe morbidity was 7% and 11%, respectively [5].

However, there was no standard definition for severe acute morbidity of abortion. Recently, suitable criteria to assess maternal near miss for sub-Saharan countries were adapted in harmony with the previous World Health Organization near-miss criteria [9].

## 2. Methods

### 2.1. Study Design

An institution-based cross-sectional study was conducted.

### 2.2. Study Area and Period

The study was conducted in Hawassa University Comprehensive Specialized Hospital from January 1 to October 30, 2019, over ten months. Hawassa is the capital city of the Southern Nation's Nationalities, and People's Region located 275 km south of Addis Ababa, the capital city of Ethiopia. The city has two governments (one general and another comprehensive) and 4 private primary hospitals and a number of public and private clinics and pharmacies. Hawassa Referral Hospital was established and started its full function in 2005. It is one of the referral hospitals in the region serving as a teaching hospital for Hawassa College of Medicine and Health Sciences. The hospital has 8 departments. Gynecology and Obstetrics is one of the departments where laboring, other obstetrics, and gynecologic conditions of mothers are followed and managed accordingly. The hospital has a catchment population of about 18 million people. According to 2016/2017 HIMS, there were about 106,012 and 11,103 patients of all types per year as outpatient and inpatient, respectively. From this report, 243 patients came with emergency abortion complications, 63 patients for safe termination, and 50 patients for suspected molar pregnancy were managed. There are four beds reserved mainly for abortion cases at gynecologic OPD. Gynecology and Obstetrics Department has one gynecologic oncologist, eight gynecologists and obstetricians, four general practitioners, and 93 midwives. There are also residents, interns, and master students working in the department.

### 2.3. Source Population

The source population is all women who came for abortion or postabortion care to HUCSH in the specified time frame.

### 2.4. Study Population

All women who were managed for clinical diagnosis of abortion-related complication at gynecologic OPD and wards during the study period were included.

### 2.5. Inclusion and Exclusion Criteria

#### 2.5.1. Inclusion Criteria

All admitted patients with a clinical diagnosis of abortion or suspected molar pregnancy (GTD) and willing to participate in the study from January 1 to October 30, 2019, were included.

#### 2.5.2. Exclusion Criteria

Those diagnosed with threatened abortion and clinically diagnosed or pathologically confirmed molar or gestational trophoblastic neoplasm at admission were excluded.

### 2.6. Sample Size Determination

A single population proportion formula was used to calculate the sample size for the magnitude of severe acute maternal morbidity (SAMM). To determine the minimum sample size for this objective (SAMM), the study considered 11% prevalence of severe morbidity obtained from a previous study in Ethiopia [5], at 95% certainty and 5% margin of error. Similarly, 16% proportion from the Zambian study was taken to determine the sample size for the magnitude of maternal near miss (MNM) related to abortion [9]. Adding 10% contingency to increase power and compensate for possible nonresponse resulted 165 and 227 samples for the magnitude of SAMM and MNM, respectively.

The sample size was calculated for associated factors with severe acute maternal morbidity related to abortion from a previous study using Epi Info version 7 statistical software [10] (Table 1).

Adding 10% contingency results minimum sample size 317.

Finally, the sample size calculated for the magnitude of SAMM and MNM was smaller than the sample size calculated for associated factors. Thus, the sample size for data collection was 317.

### 2.7. Sampling Technique

All patients who came to this tertiary hospital for abortion and postabortion care and fulfilling the inclusion criteria in the time frame were included conveniently.

### 2.8. Variables

#### 2.8.1. Independent Variables

- Sociodemographic conditions
- Reproductive characteristics
- Type of abortion diagnosis at arrival
- Interference for termination
- Mode of termination
- Logistical factors
- Referral status
- Duration of delay before arrival

#### 2.8.2. Dependent Variables

- Severe acute maternal morbidity
- Maternal near miss

### 2.9. Definition of Terms

Abortion is the termination of pregnancy before age of viability (before 28 weeks and less than 1 kg in the Ethiopian context).

Maternal near miss (MNM) refers to a woman who nearly died but survived a complication that occurred during pregnancy, childbirth, or within 42 days of termination of pregnancy.

Oliguria is urinary output < 30 ml/h for 4 h or <400 ml/24 h.

Persistent severe hypotension is defined as a systolic BP < 90 mmHg for ≥60 min with a pulse rate of at least 120 despite aggressive fluid replacement (>2 litter).

Sepsis is defined as a clinical sign of infection and 3 of the following: temp > 38°C or <36°C, respiration rate > 20/min, pulse rate > 90/min, and WBC > 12,000 or <4,000.

### 2.10. Operational Definition

GA: if the client does not remember her LNMP or has no any early milestone, gestational age will be taken in months as patient's description (first trimester ≤ 3 months, second trimester > 3 months).

Induced abortion: any deliberate attempt to initiate termination of the pregnancy by anyone before viability.

Long hospital stay: if the patient stays more than 24 hours.

Maternal death: death of pregnant women due to abortion and its complication after admission to the hospital and before discharge.

Maternal near miss: abortion or suspected GTD complicated with one or more of the following: RR < 6 or >40 breaths per minute, oxygen saturation < 90%, persistent severe hypotension, generalized peritonitis/sepsis/septic shock, organ dysfunction, DIC, GTD with anemia, transfusion ≥ 2 units, or laparotomy.

Mild complication (all criteria fulfilled): temperature < 37.3 degree Celsius, no signs of infection, and no system or organ failure.

Moderate complication (at least one): offensive products of conception, temperature 37.3-37.9 degree Celsius, localized peritonitis (tender uterus), anemia (Hgb > 7 mg/dl), and no need of transfusion.

Nonsevere complication: abortion with mild or moderate complications.

Organ dysfunction: when serum creatinine > 3.5 mg/dl, aspartate aminotransferase or alanine aminotransferase more than twice the upper limit.

Own business: housewife, farmer, or merchant.

Severe acute maternal morbidity: abortion or suspected GTD complicated with one or more of the following: temperature ≥ 38°C, pulse rate ≥ 120 beats/minute, shock (blood pressure < 90/60 mmHg), need for blood transfusion (≥1 unit), foreign body or mechanical injury on evacuation, or any of the above maternal near-miss complications. Here, it can be used interchangeably with severe complication/maternal outcome.

Severe complication: severe morbidity or death.

Spontaneous abortion: onset of expulsion of conceptus content spontaneously, as the patient's description.

Suspected molar pregnancy: abortion or postabortion cases with an impression of partial mole or passage of vesicles which is not witnessed by a health professional.

Residual sickness: a patient discharged with a sign/symptom of anemia.

Urban/rural residency: as the description of the patients.

### 2.11. Data Collection Tools and Procedures

Data was collected by using prospective morbidity methodology [11], which was developed by the WHO task force and the investigator reviewed data collection instruments from Kenya, Cambodia, Malawi, and more from Ethiopia [5, 12–14]. Then, an anonymous structured English questioner was prepared including sociodemographic characteristics, reproductive characteristics, clinical presentations, referral status, logistical factors, and severe acute abortion-related complication information from clinical features, investigations, managements, referral papers, hospital stay, and status at discharge.

### 2.12. Data Quality Management

The data quality assured by that the questionnaire was pretested on 10 cases at gynecologic OPD abortion clinic which were not involved in the study and was modified for inconveniences. Along with that, the collectors were trained for 3 days well before being involved in the collecting processes. The data was collected by trained year one residents during working hours and by trained midwives during duty hours. The information documented by the most senior attending physician on the patient's chart and postabortion care file was taken. Additional information was taken from patients or guardians for minors after written consent. There was already a separate room prepared for family planning privately which was used for this purpose during discharge. The data collectors were contacted by supervisors on daily basis in case of any problems and difficulties. The completeness of the data was checked by the principal investigator.

### 2.13. Data Processing and Analysis

The collected data was checked, cleaned, and entered into SPSS version 20 for further data cleaning and analysis. Frequency distributions were obtained to check for data entry error (missing/unrecognized values and codes). Descriptive analysis was done on the sociodemographic and reproductive characteristics, signs of severe morbidity, near miss, and outcomes of abortion. Bivariate and multivariable logistic analysis was also done to see the relationship between the dependent and independent variables. Variables with *p* value ≤ 0.2 in bivariate analysis, not collinear, were entered into multiple logistic regressions to assess the net effect by controlling confounders. The strength of association is presented by odds ratio and 95% confidence interval. *p* value < 0.05 was used as a cut-off point to determine a statistically significant association. Finally, descriptive statistics, tables, graphs, means, and frequency distribution were used to present the information.

## 3. Results

There were 345 admissions with the diagnosis of abortion and postabortion complication during the study period. Information was collected from 337 abortion and postabortion care patients giving a response rate of 97.7%. Three patients disappeared from the postabortion clinic before collection of the questioner, and five patients were not willing for the interview. The age of patients ranged from 13 to 50 years with a mean age of 25.27 ± 6.9 years. The peak age group was 18-24 years, accounting for 42.4%.

Women who came from the urban area were 202 (59.9%). About half of patients (52.8%) are protestant by religion followed by Islam (27.6%). Most of the patients are married 242 (71.8%).

About one-third (31.5%) had no education, 35.9% had elementary education, and 14.8% are college graduates. Housewives accounted for 154 (45.7%) of the patients, and only 29 (8.6%) are government employees (Table 2).

The mean gravidity was 3.1 ± 2.67, ranging from 1 to 15 pregnancies. A fourth (27.3%) of the women had had at least one previous abortion. When they conceive their current pregnancy, 91 (27%) women were using some form of contraceptive. More than half (62.9%) of the clients' gestational age was in the second trimester, of which 22.3% were more than 20 weeks (Table 3).

Vaginal bleeding was the commonest presenting symptom (54.3%) followed by abdominal pain (4.2%), fever (3%), and vaginal discharge (2.7%). Women who came for safe termination were 93 (27.6%). Only 25.5% of those symptomatic patients present to the hospital within 12 hours of manifestation of abortion complications.

As for the patients' explanation and documentations from referral papers, 58.5% were spontaneous, 38% induced by trained professionals, and 3.6% (twelve patients) were self-induced or by untrained professionals. Near half (48.1%) of the patients came directly from home, 45.7% referred from other public institutions, and 6.2% from private institutions.

During admission and/or their stay in the hospital, 120 (35.6%) had at least one severe complication characterized by physical findings, investigations, and management interventions. From physical findings, shock (6.8%), pulse rate > 119 beats/minute (17.8%), temperature > 37.9 degree Celcius (4.2%), respiratory rate > 40 breaths/minute 3 (0.9%), and sepsis/septic shock (6.2%) were found. Investigation findings include hemoglobin < 7 mg/dl (20.5%), disseminated intravascular coagulopathy (1.5%), and organ dysfunction (1.5%). As an intervention, the study found laparotomy (1.5%) and at least one unit of blood was transfused (24.9%) (Table 4).

**Figure 1:**
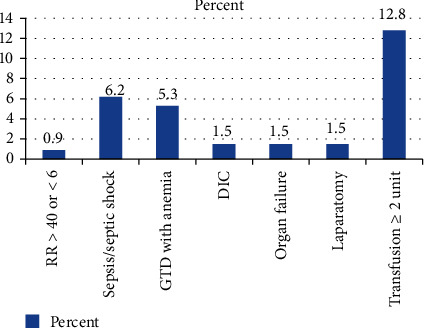
Percentage distribution of maternal near miss among postabortion care, at HUCSH, Ethiopia, from January 1 to October 30, 2019.

Maternal near miss was found in 59 (17.7%) of the patients. This include respiratory rate > 40 breaths/minute, sepsis/septic shock, DIC, organ dysfunction, and laparotomy. Additionally, 12.8% of patients who were transfused at least two units of blood and/or suspected GTD with anemia (5.3%) were included (Figure 1).

**Figure 2:**
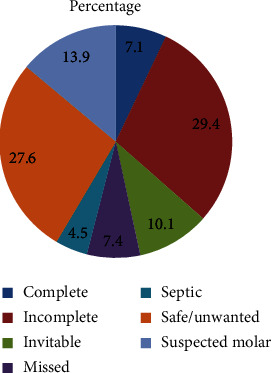
Percentage distribution of women seeking abortion care, by type of clinical diagnosis at arrival, at HUCSH, Ethiopia, from January 1 to October 30, 2019.

The commonest type of clinical diagnosis was incomplete abortion (29.4%) followed by safe/unwanted pregnancy (27.6%) who come for termination for different reasons (Figure 2).

Among all admissions, 36.5% had medical termination, 38.3% manual vacuum aspiration, 3% sharp curettage, and 9.8% electrical vacuum aspiration (Table 5).

Most patients (88.5%) were counseled about options of modern postabortion contraception. Near half (48.4%) of them accepted, and the others refused.

The mean duration of hospital stay was 45.86 ± 53.47 hours with a range of 2-576 hours, and 53.4% stayed more than 24 hours.

Majority of patients discharged improved (92.6%) and the others with residual sickness (6.2). There were also four deaths (1.2%).

All candidate variables in the chi-square test were computed with binary logistic regression. In the bivariable analysis, age, residence, religion, marital status, education, occupation, interference, gestational age, gravidity, duration of delay, referral status, mode of termination, and diagnosis type of abortion at arrival were variables with *p* values ≤ 0.2 (Table 6).

However, in multiple variable regression analysis, only education, gestational age, gravidity, delayed presentation, and type of abortion remain significantly associated with severe acute maternal morbidity at *p* value less than 0.05. Presumed correlation was checked if educational level affects gestational age and duration of delay to seek health care service for their condition but found insignificant with correlation coefficient (*r*) = 0.022, *p* value = 0.683 and *r* = 0.035, *p* value = 0.556, respectively. Women who had no education were 3 times (AOR: 3.02; 95% CI 1.24-7.33) more likely to develop severe acute maternal morbidity than those who had some primary and above education. Women whose pregnancy was in the second trimester had 4.16 (1.89-9.14) times higher odds of having severe acute maternal complication compared to those in the first trimester of pregnancy. Delayed presentation 12 hours and more after the onset of symptoms was at least 4 times (AOR:4.32, 95% CI 1.76-10.59) higher to have severe complication compared to those who come before 12 hours. Admission diagnosis of complete and incomplete abortions was associated with 13.57 (2.31-79.57) and 4.28 (1.31-14.00) times higher odds of having severe morbidity compared with those who are admitted for termination with a diagnosis of safe/unwanted pregnancy, respectively (Table 7).

## 4. Discussions

In this study, the magnitude of severe acute maternal morbidity is 35.6% (30.6-40.4%) of which four cases (1.2%) end up in death. Two deaths were admitted to this hospital with severe anemia, complete abortion, shock, and one with additional sepsis. One maternal death was 25 years old unmarried who attempt self-induced incomplete abortion with profuse vaginal bleeding and hypovolemic shock. The fourth death was after MVA for incomplete abortion, but molar pregnancy was considered after the procedure. There was ongoing vaginal bleeding and arrested while taken to the operation theater. Historically, the gestational age for all these died cases was in second trimester. In this study, severe complication is higher than the previous study extrapolated from hospitals in this region including the current setup 30% (26-34%) [6]. It is also high when compared with the result from Tikur Anbessa Tertiary Hospital which showed 21.7% before and after the liberalization of abortion law [15]. This is also higher than the previous systematic review study, 27.9% (9.7-75%) [4]. The magnitude of SAMM in this study was lower than Cambodian and comparable with Kenyan studies which were 42% and 37.1%, respectively [10, 12]. The possible explanation for this large percentage of severe acute morbidity could be due to the decreasing stigma of abortion to seek postabortion care. The fact that this facility is the only comprehensive specialized hospital in the catchment area, patients with severe complication may be referred to this tertiary hospital from different health institutions. The most common severe complication found in this study was anemia which required blood transfusion (24.9%). This was comparable with studies from Zambia and another systematic review and meta-analysis study which was 24% and 23%, respectively [8, 9].

Severe acute maternal morbidity had an association with educational status. Patients with no education had a higher risk to develop a severe complication. It was also indicated in another study that severe abortion complication slightly decreases as the level of education increases [16]. This could be a lack of information on where to get the service for safe abortion or where to seek care for early nonsevere complications. Women who presented with second-trimester pregnancy also had a higher severe complication than those who did in the first trimester. This is as expected and in line with previous studies [10, 16]. Severe maternal morbidity was associated with a delay in seeking health care after the onset of symptom. Women who came to the health facility 12 hours and more after the onset of symptom had higher odds of severe acute maternal morbidity compared with those who presented before 12 hours, which is in line with finding from Kenya [10]. At arrival diagnosis with complete and incomplete abortions were associated with a higher risk of severe complications compared with safe/unwanted pregnancy who came directly to this hospital for induced terrmination. A possible explanation for this is that complete and incomplete abortions usually came to this hospital with a referral from other health facilities with complications for further investigations and management. On the other hand, patients who came for safe termination has a legal reason; in case of rape or incest, if the woman has physical or mental disabilities, if it is needed to preserve the woman's life or her physical health, or if she is a minor who is physically or mentally unprepared for childbirth, she will undergo termination [17].

The magnitude of maternal near miss related to abortion was found to be 17.7% (13.5-21.6%). This finding was higher than the previous systematic review finding 6.3% (1.9-33.2%) [4]. In this study, better near-miss criteria were incorporated from previous WHO and recently adapted criteria for sub-Saharan countries. The result is similar to the recent Zambian study (16%). Ethiopia like Zambia is a sub-Saharan country, considers abortion below 28 weeks, and has less restrictive law for abortion. The modified criteria for near miss is helpful to trace life-threatening complications related to abortion. If we use the WHO definition of massive transfusion (≥5 units) for near-miss criteria, the magnitude of maternal near miss will be 10.5%. In this study, though there are only four abortions related to maternal death, it was possible to trace 59 (17.7%) cases of near miss by the adapted criteria. Studies from different countries show these adapted criteria are more useful where there are low resource and blood scarcity [9]. At least 2 units of blood transfused (12.8%), sepsis/septic shock (6.2%), DIC, laparotomy, and organ failure each complicated 5 (1.5%) patients. Laparotomy was performed for two uterine perforations, one generalized peritonitis, one uterine scar dehiscence, and one suspected GTD case.

In this study, the mean age of abortion cases was 25.27 ± 6.9 years. The peak age group was 18-24 years (42.4%) which was comparable with previous studies [6, 18]. Compared with the previous study, self-induced/by untrained professional had decreased (3.6% vs. 15%) [5]. This could be due to improved awareness through different media where to access the service. Second-trimester abortion care seekers were slightly higher than the previous study at the tertiary level in this country (62.9% vs. 51%) [15].

## 5. Limitations of the Study

This study was conducted in one comprehensive specialized hospital; therefore, the findings of the study might not be representative for the general population. The other drawback is high proportion of severe acute maternal morbidity, and maternal near miss is due to referrals from different sites since this is the highest institution in the region.

## 6. Conclusions and Recommendations

The magnitude of severe acute maternal morbidity is increasing which needs further effort to eliminate unsafe abortion and its grave complications in this institution and catchment area. Lack of education, increased gestational age, and delayed presentation had increased complications associated with abortion. These problems better to be addressed by the hospital and information might be delivered to the community in collaboration with appropriate media and government policy. Severe anemia and hypovolemic shock are among expected complications leading to severe morbidity including the maternal deaths encountered in this study. Considering these risks of complication, an adequate supply of blood to blood banks is needed to halt down these common complications.

Increased severe acute maternal morbidity in uneducated, second-trimester pregnancy, or those who present delay may insight the need for further study to investigate whether these problems are related to financial, transportation, information gap, or service provider factors and to take an action accordingly.

Abortion-related near miss is better addressed by reasonable adaptation of the WHO near-miss criteria for hospitals. Unlike maternal mortality related to abortion, near-miss cases could be accessed at an institutional level. Though there is no similar study in this country, the study showed similar results with other countries which support to be done in different institutions and national level.

## Figures and Tables

**Table 1:** Sample size calculation for associated factors with severe acute maternal morbidity (SAMM) from a previous study using Epi Info version 7 statistical software.

Predictor variable	Dependent variable	CI	Power of the study	AOR	% of outcome in unexposed group	Sample size	Contingency	Total sample size
Wantedness	SAMM	95%	80%	2.03	51.4%	288	10%	317
Interference	SAMM	95%	80%	2.39	26.2%	204	10%	224

**Table 2:** Percentage distribution of women seeking abortion and postabortion care, by sociodemographic characteristics, at Hawassa Comprehensive Specialized Hospital, Ethiopia, January 1 to October 30, 2019.

Characteristics	Category	Frequency	Percent
Age groups (in years)	<18	28	8.3
	18-24	143	42.4
	25-29	89	26.4
	30-34	31	9.2
	≥35	46	13.6

Residence	Urban	202	59.9
	Rural	135	40.1

Ethnicity	Oromo	120	35.6
	Sidama	144	42.7
	Amhara	27	8
	Wolayta	18	5.3
	Others	28	8.3

Religion	Orthodox	60	17.8
	Islam	93	27.6
	Protestant	178	52.8
	Others	6	1.8

Marital status	Married	242	71.8
	Single	82	24.3
	Cohabitating	9	2.7
	Separated/divorced/widowed	4	1.2

Occupation	Housewife	154	45.7
	Merchant	44	13.1
	Farmer	13	3.9
	Student	70	20.8
	Government employee	29	8.6
	Daily laborer	22	6.5
	Others	5	1.5

Educational status	None	106	31.5
	Some primary	121	35.9
	Some secondary	60	17.8
	Postsecondary	50	14.8

**Table 3:** Reproductive profile and other characteristics of respondents, at Hawassa Comprehensive Specialized Hospital, Ethiopia, January 1 to October 30, 2019.

Characteristics	Variable	Frequency	Percent
Gravidity	1	132	39.2
	2	47	13.9
	3	55	16.3
	≥4	103	30.6

Previous abortion	At least one	92	27.3
	No	245	72.7

Was using contraceptive	Yes	91	27
	No	246	73

Gestational age	First trimester	125	37.1
	Second trimester	212	62.9

Symptom/reason	Vaginal bleeding	183	54.3
	Fever	10	3
	Vaginal discharge	9	2.7
	Abdominal pain	14	4.2
	Passage of tissue	7	2.1
	Loss of consciousness	1	0.3
	Unwanted pregnancy	93	27.6
	Others	20	5.9

Delay after symptom	<12	86	25.5
	12-24 hrs	73	21.7
	>24 hrs	80	23.7

Symptom onset	Spontaneous	197	58.5
	Induced by trained professional	128	38
	Self-induced/untrained one	12	3.6

Referral status	From home	162	48.1
	From other public institution	154	45.7
	From private institution	21	6.2

**Table 4:** Percentage distribution of severe acute maternal morbidity among abortion and postabortion care, at HUCSH, Ethiopia, January 1 to October 30, 2019.

SAMM	Frequency	Percent
BP < 90/60	23	6.8
PR > 119	60	17.8
*T*° > 37.9	14	4.2
RR > 40 or <6	3	0.9
Sepsis/septic shock	21	6.2
Hgb < 7	69	20.5
DIC	5	1.5
Organ failure	5	1.5
Laparotomy	5	1.5
Transfusion	84	24.9

**Table 5:** Percentage distribution of women seeking abortion care, by received selected type of care and condition during discharge, at HUCSH, Ethiopia, from January 1 to October 30, 2019.

Intervention		Frequency	Percent
Mode of termination	Medical termination	123	36.5
	Curettage	10	3.0
	MVA	129	38.3
	Spongy forceps	4	1.2
	EVA	33	9.8
	Not done/not mentioned	38	11.3
Postabortion family planning	Offered and accepted	163	48.4
	Offered but refused	135	40.1
	Not offered	30	8.9
IV antibiotics	Yes	52	15.4
	No	285	84.6
Condition at discharge	Improved	312	92.6
	Discharged with residual sickness	21	6.2
	Died	4	1.2

**Table 6:** Bivariable logistic regression analysis showing factors associated with severe acute maternal morbidity related to abortion, at Hawassa University Comprehensive Specialized Hospital from January 1 to October 30, 2019.

Variables		Outcome (*N* = 337)		*p* value	COR (95% CI)
		Nonsevere (*n* = 217)	Severe (*n* = 120)		
Age category	(ref = <18)	19	9	0.200	1.00
	18-29	175	90	0.847	1.09 (0.47-2.54)
	>29	23	21	0.194	1.93 (0.72-5.18)
Residence	(ref = urban)	143	59		1.00
	Rural	74	61	0.003	2.00 (1.26-3.15)
Religion	(ref = orthodox)	41	19	0.009	1.00
	Islam	47	46	0.031	2.11 (1.07-4.17)
	Protestant	126	52	0.720	0.89 (0.47-1.68)
	Others	3	3	0.372	2.16 (0.340-11.70)
Marital category	(ref = married)	142	100		1.00
	Currently not married	75	20	0.001	0.38 (0.22-0.66)
Education category	(ref = educated)	48	59		1.00
	Uneducated	169	62	<0.001	3.3 (2.04-5.33)
Occupation category	(ref = own business)	122	89	0.009	1.00
	Student	50	20	0.044	0.55 (0.31-0.99)
	Government employee	22	7	0.069	0.44 (0.18-1.07)
	Daily laborer and others	23	4	0.010	0.24 (0.08-0.71)
Gravidity category	(ref = 1)	92	40	0.001	1.00
	2	27	20	0.129	1.70 (0.86-3.39)
	3	45	10	0.091	0.51 (0.23-1.11)
	≥4	53	50	0.005	2.17 (1.27-3.70)
Onset category	(ref = spontaneous)	115	82		1.00
	Induced	102	38	0.007	0.52 (0.32-0.83)
Gestational age	(ref = <first trimester)	92	23		1.00
	Second trimester	125	87	0.007	1.94 (1.20-3.14)
Delay category	(ref = <12 hours)	70	16		1.00
	≥12 hours	103	97	<0.001	4.12 (2.24-7.58)
Referral category	(ref = not referred)	127	35		1.00
	Referred	90	85	<0.001	0.29 (0.18-0.47)
Diagnosis category	Safe/unwanted pregnancy	76	17	<0.001	1.00
	Complete abortion	7	17	<0.001	10.86 (3.89-30.27)
	Incomplete abortion	61	38	0.002	2.78 (1.43-5.41)
	Inevitable abortion	33	1	0.057	0.13 (0.02-1.06)
	Missed abortion	20	5	0.845	1.12 (0.37-3.40)
	Suspected molar pregnancy	20	27	<0.001	6.04 (2.76-13.19)
Intervention category	(ref = medical)	96	27	<0.001	1.00
	Surgical	102	74	0.001	3.56 (1.65-7.64)
	Not done/explained	19	19	0.371	1.38 (0.68-2.78)

COR = crud odds ratio, CI = confidence interval.

**Table 7:** Factors associated with abortion complication severity; result from bivariable (I) and multivariate (II) logistic regression analysis, at Hawassa University Comprehensive Specialized Hospital from January 1 to October 30, 2019.

Variables		Outcome (*N* = 337)		COR (95% CI): (I)	AOR (95% CI): (II)
		Nonsevere (*n* = 217)	Severe (*n* = 120)		
Age category	(ref = <18)	19	9	1.00	1.00
	18-29	175	90	1.09 (0.47-2.50)	0.50 (0.09-2.79)
	>29	23	21	1.93 (0.72-5.18)	0.24 (0.03-1.95)
Residence	(ref = urban)	143	59	1.00	1.00
	Rural	74	61	2.00 (1.26-3.15)	1.27 (0.56-2.90)
Marital category	(ref = married)	142	100	1.00	1.00
	Currently not married	75	20	0.38 (0.22-0.66)	0.27 (0.06-1.11)
Education category	(ref = educated)	48	59	1.00	1.00
	Uneducated	169	62	3.3 (2.04-5.33)	3.02^∗^ (1.24-7.33)
Occupation category	(ref = own business)	122	89	1.00	1.00
	Student	50	20	0.55 (0.31-0.99)	4.39 (0.92-20.99)
	Government employee	22	7	0.44 (0.18-1.07)	0.88 (0.22-3.50)
	Daily laborer and others	23	4	0.24 (0.08-0.71)	0.23 (0.03-1.74)
Gravidity category	(ref = 1)	92	40	1.00	1.00
	2	27	20	1.70 (0.86-3.39)	0.57 (0.19-1.68)
	3	45	10	0.51 (0.23-1.11)	0.19^∗^ (0.05-0.71)
	≥4	53	50	2.17 (1.27-3.70)	1.71 (0.55-5.35)
Onset category	(ref = spontaneous)	115	82	1.00	1.00
	Induced	102	38	0.52 (0.32-0.83)	1.48 (0.56-3.88)
Gestational age category	(ref = <first trimester)	92	23	1.00	1.00
	Second trimester	125	87	1.94 (1.20-3.14)	4.16^∗∗∗^ (1.89-9.14)
Delay category	(ref = <12 hours)	70	16	1.00	1.00
	≥12 hours	103	97	4.12 (2.24-7.58)	4.32^∗∗^ (1.76-10.59)
Referral category	(ref = not referred)	127	35	1.00	1.00
	Referred	90	85	0.29 (0.18-0.47)	1.49 (0.67-3.33)
Diagnosis category	(ref = safe/unwanted pregnancy)	76	17	1.00	1.00
	Complete abortion	7	17	10.86 (3.89-30.27)	13.57^∗∗^ (2.31-79.57)
	Incomplete abortion	61	38	2.78 (1.43-5.41)	4.28^∗^ (1.31-14.00)
	Inevitable abortion	33	1	0.13 (0.02-1.06)	0.14 (0.01-1.58)
	Missed abortion	20	5	1.12 (0.37-3.40)	0.70 (0.13-3.763
	Suspected molar pregnancy	20	27	6.04 (2.76-13.19)	2.8 (0.46-17.23)
Intervention category	(ref = medical)	96	27	1.00	1.00
	Surgical	102	74	3.56 (1.65-7.65)	1.22 (0.50-3.00)
	Not done/explained	19	19	1.38 (0.68-2.78)	5.43 (0.81-36.43)

COR = crude odds ratio; AOR = adjusted odds ratio; CI = confidence interval. ^∗^Statistically significant at *p* < 0.05, ^∗∗^*p* < 0.01, and ^∗∗∗^*p* < 0.001.

## Data Availability

The datasets generated during the study are available from the author and could be delivered upon reasonable request.

## Abbreviations

AOR: Adjusted odds ratio
CI: Confidence interval
DIC: Disseminated intravascular coagulopathy
GA: Gestational age
GTD: Gestational trophoblastic disease
HUCSH: Hawassa University Comprehensive Specialized Hospital
LNMP: Last normal menstrual period
MNM: Maternal near miss
SAMM: Severe acute maternal morbidity
OPD: Outpatient department
SPSS: Statistical Package for Social Science
WHO: World Health Organization.
